# *In vitro* Activity of Linezolid in Combination with Photodynamic Inactivation Against *Staphylococcus aureus* Biofilms

**Published:** 2017

**Authors:** Nasim Kashef, Mahboobeh Akbarizare, Mohammad Reza Razzaghi

**Affiliations:** 1. Department of Microbiology, Faculty of Biology, College of Science, University of Tehran, Tehran, Iran; 2. Department of Microbiology, Faculty of Biology, College of Science, University of Tehran, Tehran, Iran; 3. Laser Application in Medical Sciences Research Center, Shahid Beheshti University of Medical Sciences, Tehran, Iran

**Keywords:** Antibiotic therapy, Biofilm, Photodynamic inactivation, *Staphylococcus aureus*

## Abstract

**Background:**

Biofilm infections are a major challenge in medical practice. Bacteria that live in a biofilm phenotype are more resistant to both antimicrobial therapy and host immune responses compared to their planktonic counterparts. So, there is need for new therapeutic strategies to combat these infections. A promising approach [known as Photodynamic Inactivation (PDI)] to kill bacteria growing as biofilms uses light in combination with a photosensitizer to induce a phototoxic reaction which produces reactive oxygen species that can destroy lipids and proteins causing cell death. PDI does not always guarantee full success, so, combination of PDI with antibiotics may give increased efficiency. This study aimed to determine if PDI was effective in the eradication of *Staphylococcus aureus* (*S. aureus*) biofilms in combination with linezolid.

**Methods:**

The susceptibility of biofilm cultures of three *S. aureus* strains to Methylene Blue (MB) and Toluidine Blue O (TBO)-mediated PDI was determined alone and in combination with linezolid.

**Results:**

Bactericidal activity (≥3 log_10_ reduction in viable cell count) was not achieved with MB/TBO-PDI or antibiotic treatment alone. When antibiotic treatment was combined with TBO-PDI, a greater reduction in viable count than antibiotic alone was observed for two strains.

**Conclusion:**

This study showed that although TBO-PDI did not have good bactericidal activity against *S. aureus* biofilms; it increased the antimicrobial activity of linezolid against these bacteria.

## Introduction

Biofilms are microbial communities that are enclosed in a matrix of exopolysaccharide 1. This gelatinous matrix allows the growing biofilm to develop a three-dimensional structure that secures long term survival of the bacteria and makes them less susceptible to antimicrobial agents and host immune response 2. Biofilms can persist in 20 to 1000 times the concentrations of drugs that inhibit planktonic bacteria and this is due to restricted antibiotic diffusion through the matrix, slow growth rates, and induction of a resistant phenotype 3,4. Biofilms are responsible for a large number of persistent human infections such as infections of the urinary tract, middle ear, sinuses and wounds 2,5.

One promising solution to the problem posed by the reduced susceptibility of biofilms to antibiotics is Photodynamic Inactivation (PDI) 6. PDI is a process in which microorganisms are treated with a Photosensitizer (PS) and then irradiated with appropriate wavelength of visible light. The photochemical reactions generate cytotoxic Reactive Oxygen Species (ROS) which are able to exert bactericidal effect 7.

Several researches have indicated that the phototoxic effects of the PSs for eradication of biofilms are different from those of planktonic cultures 8–11. One important reason is that the penetration of the PS into the biofilms is affected by the presence of extracellular polymeric substance in the biofilms, so the phototoxic effect of the PS is reduced 9.

Some researchers have reported the effects of PDI followed by antibiotic treatment on pathogenic bacteria 12–15. According to these studies, combinations of PDI with antibiotics may give increased efficacy; so, the aim of this study was to determine whether an increased effect on eradication of *Staphylococcus aureus* (*S. aureus*) biofilms was evident if PDI was used followed by linezolid. Linezolid is an oxazolidinone antibiotic with activity against gram-positive organisms, including vancomycin-resistant enterococci, multidrug-resistant pneumococci, and methicillin-resistant *S. aureus* 16.

## Materials and Methods

### Bacterial strains and culture conditions

The microorganisms used in this study were *S. aureus* UTMC 1440, *S. aureus* UTMC 1454 and *S. aureus* ATCC 25923. *S. aureus* UTMC 1440 and *S. aureus* UTMC 1454 had previously been isolated from chronic diabetic foot ulcers and characterized by conventional biochemical tests using standard methods. Bacteria were grown overnight in tryptic soy broth (Merck) under aerobic conditions at 37°*C*.

### Assessment of biofilm formation

Assessment of biofilm formation was performed according to Sharma *et al* study 17.

### Photosensitizer and light source

Methylene Blue (MB) (Sigma, UK) and Toluidine Blue O (TBO) (Sigma St. Louis, MO, USA) solutions were prepared fresh for each experiment in sterile PBS (pH=7.4), filter-sterilized and kept in the dark.

A 35 *mW* diode laser (Lasotronic-Switzerland, emitting light with a wavelength of 660 *nm*, fluence rate of 91 *mW/cm*^2^) and a 10 *mW* diode laser (MUSTANG-Russia, emitting light with a wavelength of 630 *nm*, fluence rate of 26 *mW/cm*^2^) was used for irradiation of samples incubated with MB and TBO, respectively.

Energy density delivered to each well was calculated using the following formula:

$$\text{Energy}\;\text{density}\;(\text{J}/cm^{2})=\text{Power}\;(W)\times \text{time}\;(s)/\text{area}\;\text{of}\;\text{each}\;\text{well}\;(cm^{2})$$

The diameter of each well was 7 *mm*.

### Susceptibility of biofilm-grown bacteria to PDI

Overnight cultures of *S. aureus* strains were diluted at 1:100, in TSB containing 0.25% glucose. 100 *μl* of the diluted bacterial suspensions and 100 *μl* of fresh TSB were inoculated into 96-well flat-bottomed polystyrene microplates (SPL, Korea), and incubated for 18 *hr* at 37°*C*. Following incubation, planktonic cells were removed from the microwells, 200 *μl* of MB/TBO at final concentration: 5, 50, and 500 *μg/ml* was added to each well and the plates were incubated in the dark for 10 *min* at room temperature. Following incubation, MB/TBO was removed from the microwells and the biofilms were washed with fresh PBS. MB/TBO-treated biofilms were irradiated with red light for 10 *min* (light dose: 54.6 J/*cm*^2^ and 15.6 J/*cm*^2^, respectively). Treated and untreated samples were serially diluted, plated on nutrient agar plates, and incubated for 24 *hr* at 37°*C* in the dark.

PDI experiments were also repeated with MB/TBO (final concentration: 500 *μg/ml*) at various incubation times (10, 15 and 20 *min*). All experiments were done in triplicate.

### Susceptibility of biofilm-grown bacteria to PDI and linezolid in combination

Linezolid powder (Pharmacia, USA) was a gift from Dr. Alireza Foroumadi (Faculty of Pharmacy, Tehran University of Medical Sciences, Tehran, Iran).

Biofilms were allowed to form as described above and incubated with MB/TBO at a final concentration of 500 *μg/ml* in the dark and at room temperature for 10 *min*. MB/TBO-treated biofilms were irradiated with red light for 10 *min* (light dose: 54.6 J/*cm*^2^ and 15.6 J/*cm*^2^, respectively). 200 *μl* of antibiotic was added to each well at a fixed concentration of 1600 *mg/L* (400 times more than linezolid Minimum Inhibitory Concentration (MIC) values for these strains in planktonic forms) and plates were incubated for 24 *hr* at 37°*C* in the dark. Supernatant was removed from the micro-wells. Treated and untreated samples were diluted, plated on nutrient agar plates, and incubated for 24 *hr* at 37°*C*. All experiments were done in triplicate. PDI/antibiotic combinations that reduced the original inoculum by ≥3 log_10_ *cfu/mL* were considered bactericidal 18.

### Statistical analysis

Values were expressed as log_10_ means±standard deviation. Comparisons between means of groups were analyzed using the One-Way ANOVA and Post Hoc Bonferroni tests. P<0.05 was considered statistically significant.

## Results

### Biofilm formation

As indicated by the results of the crystal violet assay (Table 1), each three *S. aureus* strains produced biofilms [Optical Density (OD) >0.17].

**Table 1.** Biofilm formation ability of two clinical *S. aureus* isolates and *S. aureus* (ATCC 25923)

***S. aureus* strains**	**Mean±SD (OD_490_)**
**ATCC 25923**	0.76±0.29
**UTMC 1440**	0.83±0.35
**UTMC 1454**	0.62±0.45

### Susceptibility of biofilm-grown bacteria to PDI

**Figure 1.**
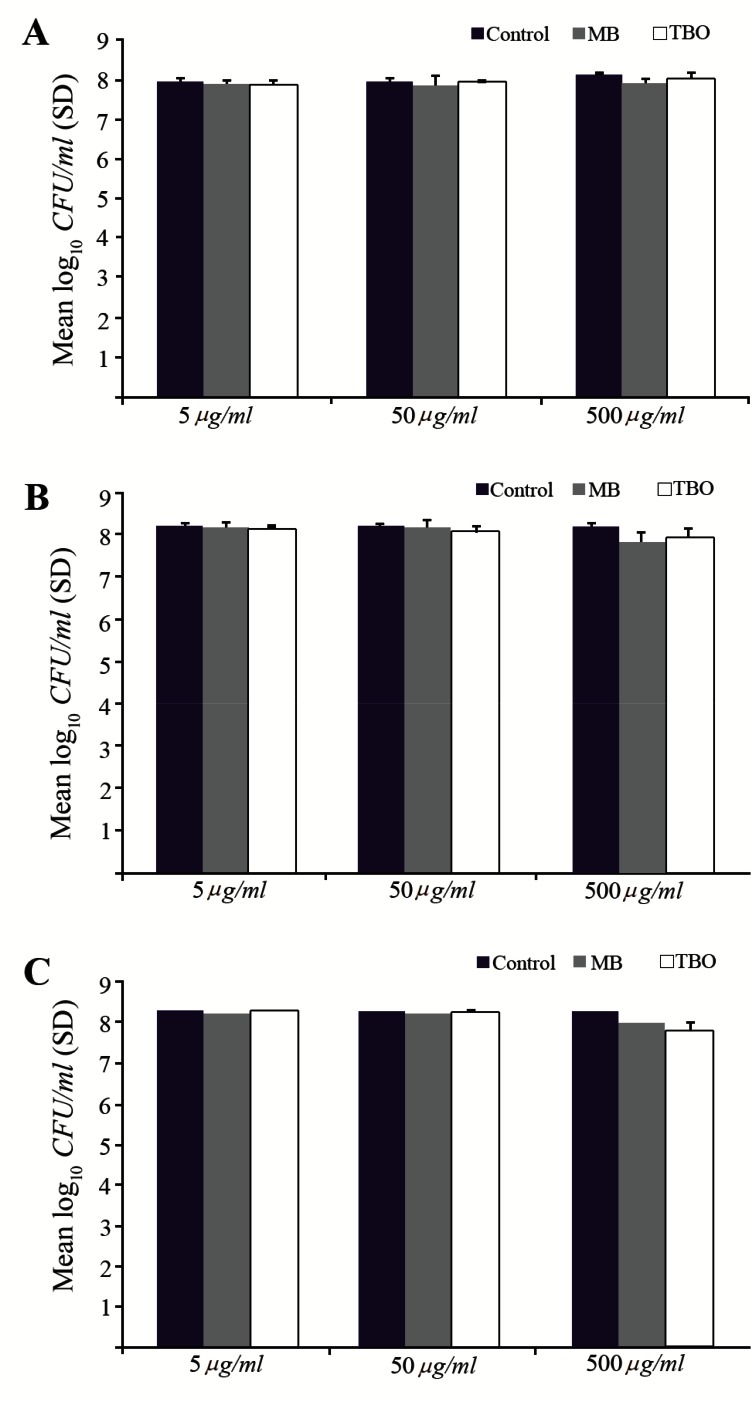
Effect of exposure to MB and TBO-PDI (diode laser, red light, light dose: 54.6 J/*cm*^2^ and 15.6 J/*cm*^2^, respectively) at a range of PS concentrations on killing of biofilm-grown strains. A) *S. aureus* (ATCC 25923), B) *S. aureus* (UTMC 1440), C) *S. aureus* (UTMC 1454).

**Figure 2.**
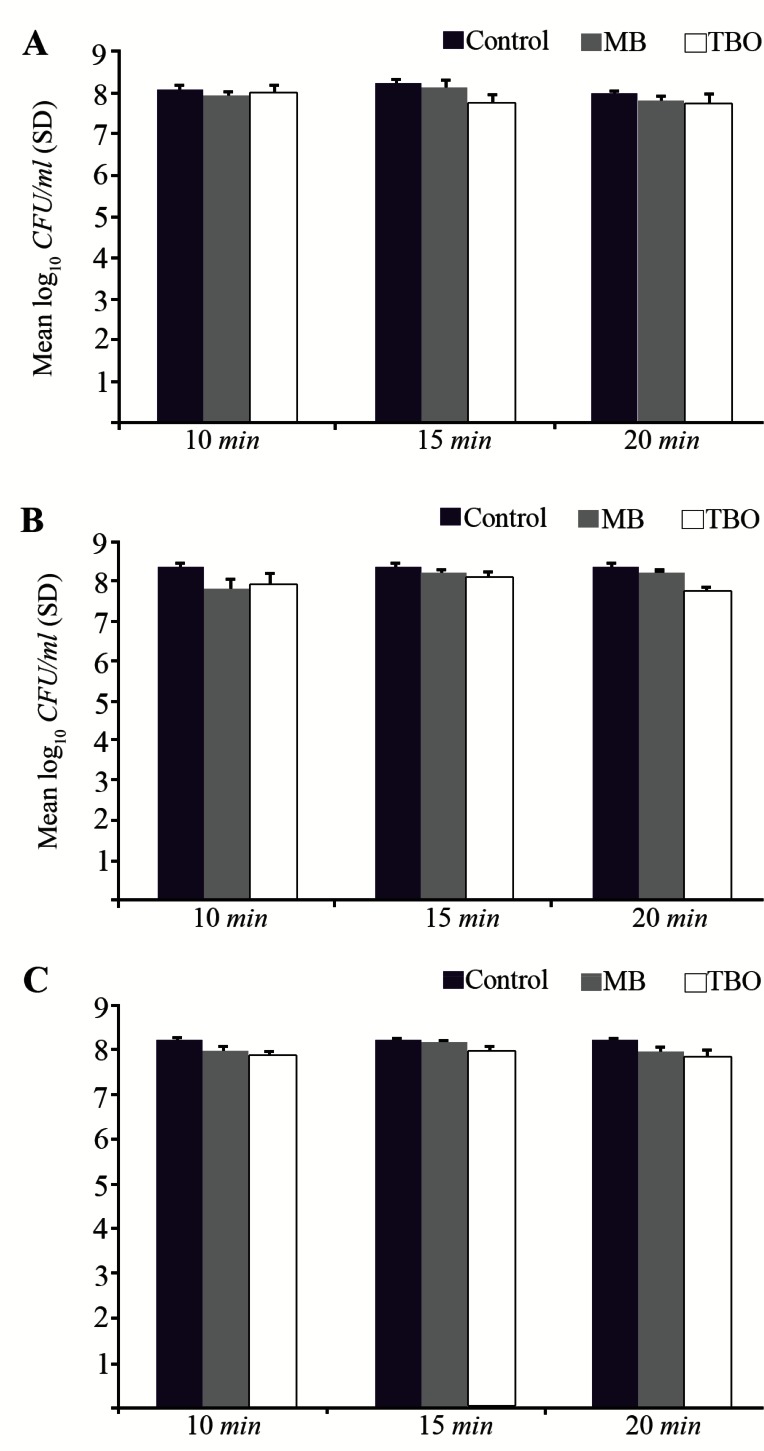
Effect of exposure to MB and TBO-PDI (diode laser, red light, light dose: 54.6 J/*cm*^2^ and 15.6 J/*cm*^2^, respectively) at a range of incubation time of PSs (500 *μg/ml*) on killing of biofilm-grown strains. A) *S. aureus* (ATCC 25923), B) *S. aureus* (UTMC 1440), C) *S. aureus* (UTMC 1454).

MB and TBO-PDI did not result in a significant reduction in viable count for any of the strains when grown in biofilms (Figure 1). Microbial reduction was not greater than 0.6 log_10_ with MB or 0.7 log10 with TBO. The survival of each three *S. aureus* strains in biofilms did not decrease with increasing PS concentrations (Figure 1) or increasing incubation time of PSs (Figure 2).

### Susceptibility of biofilm-grown bacteria to PDI and linezolid in combination

**Figure 3.**
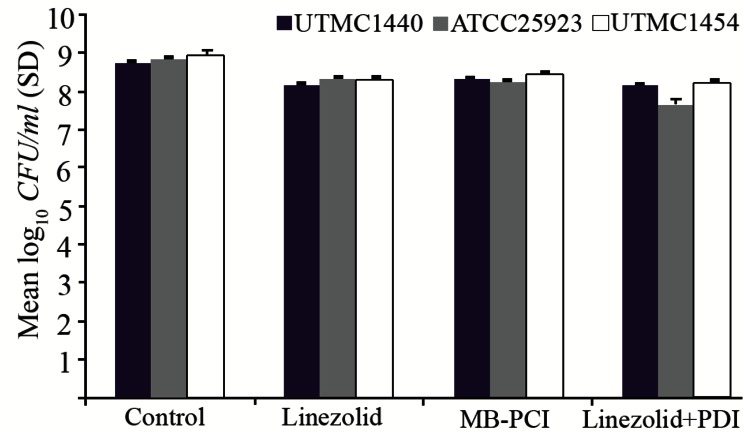
Effect of exposure to MB-PDI (diode laser, 660 *nm*, 54.6 J/*cm*^2^) and linezolid (1600 *mg/L*) on killing of biofilm-grown strains.

**Figure 4.**
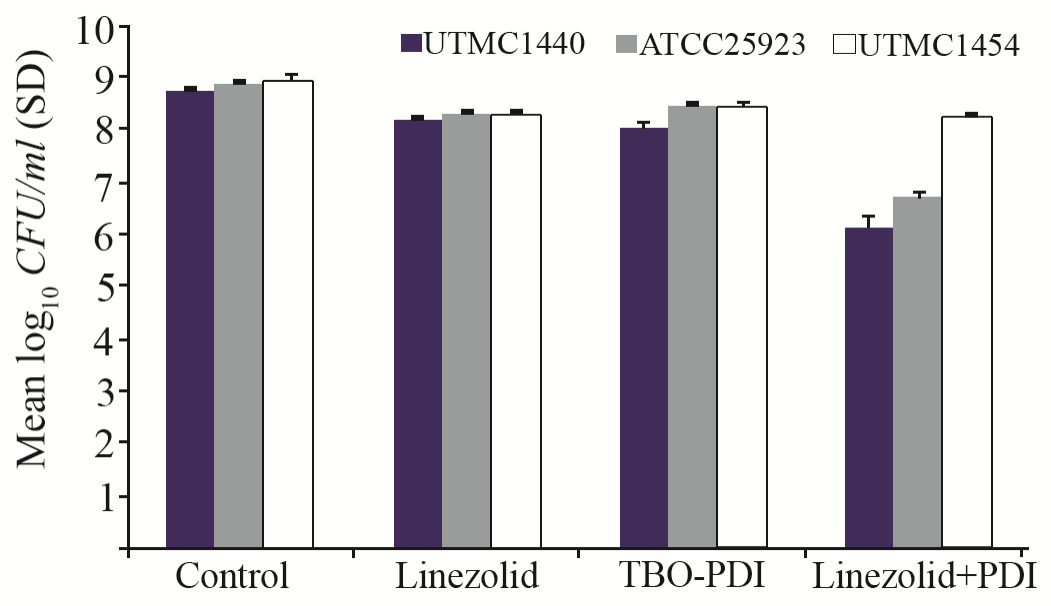
Effect of exposure to TBO-PDI (diode laser, 630 *nm*, 15.6 J/*cm*^2^) and linezolid (1600 *mg/L*) on killing of biofilm-grown strains.

Figures 3 and 4 show susceptibility of biofilm-grown bacteria to PDI and antibiotic (linezolid) in combination.

When exposed to antibiotic only, microbial reduction in comparison with the untreated control, was not greater than 0.7 log_10_ for each three strain. For *S. aureus* (ATCC 25923), the combination of MB-PDI and antibiotic resulted in a greater reduction in viable count (1.2 log_10_-unit reduction) than antibiotic alone (0.6 log_10_-unit reduction).

When antibiotic treatment was combined with TBOPDI, a greater reduction in viable count than antibiotic alone was observed for *S. aureus* (ATCC 25923), and *S. aureus* (UTMC 1440) [2.1 and 2.6 log_10_- unit reduction, respectively]. Neither linezolid nor MB/TBO-PDI exhibited bactericidal activity when used alone for 3 strains. Bactericidal activity was not achieved even with PDI-linezolid combination.

## Discussion

In the present study, MB and TBO-PDI did not result in a significant reduction (≥3 log_10_) in viable count for any of the strains when grown in biofilms. Vilela *et al* also showed that microbial reduction was not greater than 1 log_10_ with MB or TBO 19.

Poly-N-acetyl Glucosamine (PNAG) polymer is required for bacterial adherence and biofilm formation of *Staphylococcus* species 20. As PNAG is a positively charged linear homoglycan, penetration of cationic PSs such as MB and TBO could be difficult through bio-films composed of PNAG. In this study, the survival of each three *S. aureus* strains in biofilms did not decrease even with increasing PS concentrations or increasing incubation time of PSs. So, to overcome this problem, applying other PSs may improve the efficacy of PDI on *S. aureus* biofilms. Other PSs such as Aminolevulinic Acid (ALA) 21, protoporphyrin-IX 22, and chlorine e6 23 have been used to evaluate the effectiveness of PDI on *S. aureus* biofilms. In all cases, the reduction in cell survival within biofilms and the disruption of biofilms were observed.

In this study, we demonstrated that pretreatment of *S. aureus* biofilms with TBO-PDI, followed by addition of linezolid at concentration significantly below the biofilm eradication concentration values, had better effect on killing of bacteria in biofilms compared to each treatment alone. In Di Poto *et al* study, TMP-PDI-treated *S. aureus* biofilms exposed to vancomycin resulted in their almost eradication. Their study showed that PDI increased susceptibility to vancomycin and it was suggested that this was as a result of the destruction of the biofilm matrix covering the bacterial cells, making them susceptible to the antibiotic 24. Cassidy *et al* also studied the increased bactericidal effect of PDI and antibiotic treatment in combination on *Burkholderia cepacia* complex strains 14. Similarly, Dastgheyb *et al* investigated combined use of antibiotics and mesotetra (4-aminophenyl) porphine (TAPP) for treatment of *S. aureus* contamination 15.

In PDI, due to the short lifespan and limited diffusibility of singlet oxygen 25, cellular damage occurs in regions near to the PS and is not particularly targeted to structures within the bacterial cell. Therefore, the mechanism by which PDI increases linezolid susceptibility, may be due to the increased permeability of the biofilm matrix. In summary, this study showed that although TBO-PDI did not have good bactericidal activity against *S. aureus* biofilms, it increased the antimicrobial activity of linezolid against these bacteria.

## References

[1] Hall-Stoodley L Costerton JW Stoodley P. Bacterial biofilms: from the natural environment to infectious diseases. Nat Rev Microbiol 2004; 2 (2): 95– 108. 15040259 10.1038/nrmicro821

[2] Bryers JD. Medical biofilms. Biotechnol Bioeng 2008; 100 (1): 1– 18. 18366134 10.1002/bit.21838 PMC2706312

[3] El-Azizi M Rao S Kanchanapoom T Khardori N. In vitro activity of vancomycin, quinupristin/dalfopristin, and linezoid against intact and disrupted biofilms of staphylococci. Ann Clin Microbio Antimicrob 2005; 4: 2. 10.1186/1476-0711-4-2 PMC546415 15638934

[4] Mah TF O’Toole GA. Mechanisms of biofilm resistance to antimicrobial agents. Trends Microbiol 2001; 9 (1): 34– 39. 11166241 10.1016/s0966-842x(00)01913-2

[5] Kim PY Kim YS Koo IG Jung JC Kim GJ Choi MY Bacterial inactivation of wound infection in a human skin model by liquid-phase discharge plasma. PLoS ONE 2011; 6 (8): e24104. 21897870 10.1371/journal.pone.0024104 PMC3163682

[6] Wainwright M Crossley KB. Photosensitizing agents-circumventing resistance down biofilms: a review. Int Biodeter Biodegrad 2004; 53: 119– 126.

[7] Jori G. Photodynamic therapy of microbial infections: state of the art and perspectives. J Environ Pathol Toxicol Oncol 2006; 25 (1–2): 505– 519. 16566738 10.1615/jenvironpatholtoxicoloncol.v25.i1-2.320

[8] Beirão S Fernandes S Coelho J Faustino MA Tomé JP Neves MG Photodynamic inactivation of bacterial and yeast biofilms with a cationic porphyrin. Photochem Photobiol 2014; 90 (6): 1387– 1396. 25112506 10.1111/php.12331

[9] Gad F Zahra T Hasan T Hamblin MR. Effects of growth phase and extracellular slime on photodynamic inactivation of gram-positive pathogenic bacteria. Antimicrob Agents Chemother 2004; 48 (6): 2173– 2178. 15155218 10.1128/AAC.48.6.2173-2178.2004 PMC415578

[10] Lin HY Chen CT Huang CT. Use of merocyanine 540 for photodynamic inactivation of Staphylococcus aureus planktonic and biofilm cells. Appl Environ Microbiol 2004; 70 (11): 6453– 6458. 15528505 10.1128/AEM.70.11.6453-6458.2004 PMC525131

[11] Kashef N Karami S Djavid GE. Phototoxic effect of hypericin alone and in combination with acetylcysteine on Staphylococcus aureus biofilms. Photodiagnosis Photodyn Ther 2015; 12 (2): 186– 192. 25892001 10.1016/j.pdpdt.2015.04.001

[12] Chibebe Junior J Fuchs BB Sabino CP Junqueira JC Jorge AO Ribeiro MS Photodynamic and antibiotic therapy impair the pathogenesis of Enterococcus faecium in a whole animal insect model. PLoS One 2013; 8 (2): e55926. 23457486 10.1371/journal.pone.0055926 PMC3573038

[13] Barra F Roscetto E Soriano AA Vollaro A Postiglione I Pierantoni GM Photodynamic and antibiotic therapy in combination to fight biofilms and resistant surface bacterial infections. Int J Mol Sci 2015; 16 (9): 20417– 20430. 26343645 10.3390/ijms160920417 PMC4613211

[14] Cassidy CM Donnelly RF Elborn JS Magee ND Tunney MM. Photodynamic Antimicrobial Chemotherapy (PACT) in combination with antibiotics for treatment of Burkholderia cepacia complex infection. J Photochem Photobiol B 2012; 106: 95– 100. 22079165 10.1016/j.jphotobiol.2011.10.010

[15] Dastgheyb SS Eckmann DM Composto RJ Hickok NJ. Photo-activated porphyrin in combination with antibiotics: therapies against Staphylococci. J Photochem Photobiol B 2013; 129: 27– 35. 24148969 10.1016/j.jphotobiol.2013.09.006 PMC3926106

[16] Swaney SM Aoki H Ganoza MC Shinbarger DL. The oxazolidinone linezolid inhibits intiation of protein synthesis in bacteria. Antimicrob Agents Chemother 1998; 42 (12): 3251– 3255. 9835522 10.1128/aac.42.12.3251 PMC106030

[17] Sharma M Visai L Bragheri F Cristiani I Gupta PK Speziale P. Toluidine blue-mediated photodynamic effects on staphylococcal biofilms. Antimicrob Agents Chemother 2008; 52 (1): 299– 305. 17967908 10.1128/AAC.00988-07 PMC2223877

[18] Clinical and Laboratory Standards Institute publications Methods for determining bactericidal activity of antimicrobial agents; Approved Guideline. Pennsylvania: Wayne, PA; 1999 7 p.

[19] Vilela SF Junqueira JC Barbosa JO Majewski M Munin E Jorge AO. Photodynamic inactivation of Staphylococcus aureus and Escherichia coli biofilms by malachite green and phenothiazine dyes: an in vitro study. Arch Oral Biol 2012; 57 (6): 704– 710. 22208389 10.1016/j.archoralbio.2011.12.002

[20] Izano EA Amarante MA Kher WB Kaplan JB. Differential roles of poly-N-acetylglucosamine surface polysaccharide and extracellular DNA in Staphylococcus aureus and Staphylococcus epidermidis biofilms. Appl Environ Microbiol 2008; 47 (2): 470– 476. 10.1128/AEM.02073-07 PMC2223269 18039822

[21] Li X Guo H Tian Q Zheng G Hu Y Fu Y Effects of 5-aminolevulinic acid-mediated photodynamic therapy on antibiotic-resistant staphylococcal biofilm: an in vitro study. J Surg Res 2013; 184 (2): 1013– 1021. 23622723 10.1016/j.jss.2013.03.094

[22] Grinholc M Rapacka-Zdonczyk A Rybak B Szabados F Bielawski KP. Multiresistant strains are as susceptible to photodynamic inactivation as their naïve counterparts: protoporphyrin IX-mediated photoinactivation reveals differences between methicillin-resistant and methicillin-sensitive Staphylococcus aureus strains. Photomed Laser Surg 2014; 32 (3): 121– 129. 24527879 10.1089/pho.2013.3663 PMC3952657

[23] Park JH Ahn MY Kim YC Kim SA Moon YH Ahn SG In vitro and in vivo antimicrobial effect of photodynamic therapy using a highly pure chlorin e6 against Staphylococcus aureus Xen29. Biol Pharm Bull 2012; 35 (4): 509– 514. 22466554 10.1248/bpb.35.509

[24] Di Poto A Sbarra MS Provenza G Visai L Speziale P. The effect of photodynamic treatment combined with antibiotic action or host defense mechanisms on Staphylococcus aureus biofilms. Biomaterials 2009; 30 (18): 3158– 3166. 19329182 10.1016/j.biomaterials.2009.02.038

[25] Lavi A Weitman H Holmes RT Smith KM Ehrenberg B. The depth of porphyrin in a membrane and the membrane’s physical properties affect the photosensitizing efficiency. Biophys J 2002; 82 (4): 2101– 2110. 11916866 10.1016/S0006-3495(02)75557-4 PMC1302004

